# Objectified Body Consciousness, Body Image Control in Photos, and Problematic Social Networking: The Role of Appearance Control Beliefs

**DOI:** 10.3389/fpsyg.2020.00147

**Published:** 2020-02-25

**Authors:** Valentina Boursier, Francesca Gioia, Mark D. Griffiths

**Affiliations:** ^1^Department of Humanities, University of Naples Federico II, Naples, Italy; ^2^School of Social Sciences, Nottingham Trent University, Nottingham, United Kingdom

**Keywords:** appearance control beliefs, objectified body consciousness, body image, problematic social network site use, social networking, adolescence

## Abstract

At present, adolescents’ photo-taking and photo-sharing on social media represent ubiquitous practices and objectified body consciousness (OBC) might offer a useful framework to explore online self-presentation and social networking site (SNS) use. Indeed, SNS might represent a highly accessible medium for socializing with self-objectification. However, the relationship between OBC components and problematic SNS use is still understudied. The present study evaluated the previously unexplored predictive role of appearance control beliefs on problematic SNS use, testing the mediating effect of body image control in photos (BICP) across male and female groups. A total of 693 adolescents (55% females; mean age 16 years) participated in the study. Results showed the negatively predictive role of appearance control beliefs on control over body image in photos. Moreover, BICP mediated the appearance control beliefs’ negative effect on problematic SNS use in girls. The present study tested the unexplored effect of appearance control beliefs upon problematic SNS use, contributing to the OBC research field and the ongoing debate concerning predictive and protective factors in problematic SNS use.

## Introduction

In recent years, self-focused photo-taking and photo-sharing on social networking sites (SNSs) have become ubiquitous practices and objectification theory (Fredrickson and Roberts, 1997) might offer a useful framework to explore online self-presentation and SNS use (Fox and Vendemia, 2016). *Objectification theory* (Fredrickson and Roberts, 1997) provides a framework to understand the possible consequences of being female in Western societies, where female bodies are constructed as objects, looked at, commented upon, and evaluated primarily on the basis of bodily appearance (Holland and Tiggemann, 2016; Karsay et al., 2018). A close predecessor of objectification theory is the psychological construct identified in objectified body consciousness (OBC) (McKinley and Hyde, 1996). These frameworks posit that repeated objectification experiences might lead to females’ *self-objectification* and facilitate such individuals to assume and internalize an outside observer’s gaze on their physical selves (Fredrickson and Roberts, 1997; Moradi and Huang, 2008; Feltman and Szymanski, 2018). This particular view of self might lead to a form of self-consciousness in which females develop identities strongly rooted in (and defined by) their physical appearance (McKinley and Hyde, 1996; Fredrickson and Roberts, 1997; McKinley, 1999; Sinclair and Myers, 2004; Sinclair, 2005). However, an increasing number of studies have highlighted that self-objectification and consequent beliefs are also experienced by males, especially in adolescence (Moradi and Huang, 2008; Daniel and Bridges, 2010; Moradi, 2010; Vandenbosch and Eggermont, 2013; Dakanalis et al., 2015; Manago et al., 2015; Holland and Tiggemann, 2016; Karsay et al., 2018).

McKinley and Hyde (1996) operationalized OBC including three main components. The central tenet *body surveillance* represents persistent thinking and constant self-monitoring assuming an outside observer’s perspective to comply with cultural body standards and avoid negative judgments. *Body shame* arises due to the comparison with cultural standards and the perception of failure to meet them. Finally, *appearance control beliefs* refer to those beliefs that individuals are responsible for their bodily look and that, with enough effort, their physical appearance can be controlled. Typically, self-objectification and OBC have been explored in relation to consumption of traditional mass media, such as magazines, television, and films (for a review, see Grabe et al., 2008). In fact, a few studies have shown that the increasing exposure to objectifying media and images might lead to body self-objectification by individuals (e.g. Fredrickson and Roberts, 1997; Aubrey, 2006; Meier and Gray, 2014). In the past few years, the increasing popularity of social media use and the large increase in engagement of adolescents and young people in social networking (Mascheroni and Ólafsson, 2018; D’Arienzo et al., 2019; Gioia and Boursier, 2019) have led researchers to focus on SNS use as a new and highly accessible medium for socializing with self-objectification experiences and OBC (de Vries and Peter, 2013; Fardouly et al., 2015; Manago et al., 2015; Bell et al., 2018; Cohen et al., 2018; Caso et al., 2019).

The objectification theory research field (Fredrickson and Roberts, 1997) has traditionally explored the predictive role of SNS use on self-objectification experiences (Vandenbosch and Eggermont, 2012; de Vries and Peter, 2013; Fardouly et al., 2015, 2018; Manago et al., 2015; McLean et al., 2015; Holland and Tiggemann, 2016; Bell et al., 2018; Cohen et al., 2018; Feltman and Szymanski, 2018; Tiggemann and Barbato, 2018; Butkowski et al., 2019). Nevertheless, as Moradi and Huang (2008) stated, further research concerning the possible effects of OBC on subsequent outcomes is needed. Only recently, Veldhuis et al. (2018) evaluated the influence of self-objectification on SNS use, confirming the plausibility of Strelan and Hargreaves’ (2005) circle of self-objectification and, thus, the bidirectional nature of the SNS use-self-objectification pathway. In terms of OBC (McKinley and Hyde, 1996), body surveillance has been the most investigated OBC factor in association with social networking. Some studies have highlighted the strong predictive role of SNS involvement on body surveillance (Tiggemann and Slater, 2013; Fardouly et al., 2018; Feltman and Szymanski, 2018), which in turn predicts greater body shame (Manago et al., 2015; Slater and Tiggemann, 2015; Tiggemann and Slater, 2015). Veldhuis et al. (2018) hypothesized and confirmed the predictive role of body surveillance on selfie-related activities on SNSs. Few studies have explored the predictive role of adolescents’ risky sexual behaviors online concerning body surveillance (Vandenbosch and Eggermont, 2013; Doornwaard et al., 2014), and more recently, one study included appearance control beliefs to evaluate the predictive role of OBC on teenagers’ sexting for sexual purposes (Bianchi et al., 2017). However, no studies have specifically focused on the relationship between SNSs and appearance control beliefs.

Traditionally, appearance control beliefs represent a controversial and debated factor of OBC. McKinley and Hyde (1996) themselves located appearance control beliefs in a paradoxical position within OBC theory (John and Ebbeck, 2008). Even though the authors hypothesized that higher beliefs in the ability of individuals to control own appearance might contribute to them negatively experiencing their own body, their findings and several subsequent studies have reported a negative correlation or no connection between appearance control beliefs and body surveillance, body shame, and other body-related negative outcomes (i.e. eating disorder symptomatology and internalization of cultural standards of attractiveness) (McKinley and Hyde, 1996; Sinclair and Myers, 2004; John and Ebbeck, 2008; Moradi, 2010; Sinclair, 2010; Fitzsimmons-Craft et al., 2011). On the contrary, in other research, appearance control beliefs have shown significant positive association with measures of psychological well-being, body esteem, and body satisfaction (McKinley and Hyde, 1996; McKinley, 1999; Sinclair and Myers, 2004; John and Ebbeck, 2008; Crawford et al., 2009; Noser and Zeigler-Hill, 2014). Moreover, appearance control beliefs have been found to strongly and positively relate to indicators of personal agency, sense of competence, locus of control, and perceived generalized controllability over life events (McKinley and Hyde, 1996; McKinley, 1998, 1999; Sinclair and Myers, 2004; Laliberte et al., 2007; Moradi, 2010; Sinclair, 2010). On the contrary, within the addictive behaviors research field and thus from another perspective, some studies highlighted that beliefs in control over information (and perhaps also over own appearance) might promote individuals’ trust about their ability to manage it and SNSs, reducing the perception of online risks (Niemz et al., 2005; Joinson et al., 2010; Krasnova et al., 2010; Taddei and Contena, 2013). Similarly, positive metacognitions (Spada et al., 2015) have been conceptualized as specific beliefs related to a behavior as a way to control and regulate cognition and emotion. Several studies have found that these metacognitions strongly promote individuals’ engagement in Internet-related problematic behaviors (Spada et al., 2007, 2015; Casale et al., 2016, 2018; Spada and Marino, 2017). Nevertheless, within the OBC framework, the relationships between appearance control beliefs and SNS use and misuse are still unexplored.

Social networking sites are virtual communities that allow users to be not just passive receivers but also active creators of individual private or public profiles, sharing various forms of personal content, interacting with “offline” friends, meeting other people who share common interests, and viewing, commenting, and “liking” peer-generated content (e.g. Boyd and Ellison, 2007; Kuss and Griffiths, 2011b, 2017; Perloff, 2014; Holland and Tiggemann, 2016; Balakrishnan and Griffiths, 2017; Tiggemann and Slater, 2017; Boursier and Manna, 2018a; Boursier et al., 2018; Cohen et al., 2018; Veldhuis et al., 2018; Butkowski et al., 2019). However, social networking-related risks and opportunities remain a matter of scientific debate (Livingstone, 2008; Munno et al., 2017). On the one hand, SNS use could be considered as a “way of being” (Kuss and Griffiths, 2017), supporting adolescents’ need to belong and representing ideal places for their identity construction processes via a digital screen (Zhao et al., 2008; Riva, 2010; Pelosi et al., 2014; Manago et al., 2015; Boursier and Manna, 2019). On the other hand, possible social networking-related risks fuel the scientific debate about overpathologized, problematic, and potentially addictive use of SNSs (e.g. Kuss and Griffiths, 2011a, 2017; Billieux et al., 2015; Andreassen et al., 2016; Bányai et al., 2017; Franchina and Lo Coco, 2018; Kırcaburun and Griffiths, 2018). According to Kuss and Griffiths (2017), within social media and SNS research fields, unanimous agreement about terminological and operational definitions is still lacking. Within the biopsychosocial framework, some studies have utilized the six criteria of the component model of addiction (i.e. salience, mood modification, tolerance, withdrawal, relapse, and conflict) to evaluate problematic SNS use (e.g. Griffiths, 2005; Andreassen et al., 2016; Kuss and Griffiths, 2017; Monacis et al., 2017; D’Arienzo et al., 2019). On the contrary, according to a social–cognitive model, researchers have conceptualized problematic online activities in terms of difficulties in impulse control and mood regulation, subsequent negative outcomes resulting from online misuse, and preference for online social interactions, due to a perceived lack of social skills (e.g. Caplan, 2003; Baker and White, 2010; LaRose et al., 2010; Pontes et al., 2016; Casale and Fioravanti, 2017; Lee et al., 2017). In this regard, SNS use might allow young users to (i) avoid face-to-face difficulties, (ii) provide greater control over informational disclosure, and (iii) be strategic in managing own self-presentation (Casale and Fioravanti, 2017), especially through the widespread use of pictures, videos, and stories shared on SNS.

According to Feltman and Szymanski (2018), social networking use appears to be increasingly based upon the sharing of visual content that boys and girls might use as a source of comparison and information to improve their physical appearance (Rousseau et al., 2017; Franchina and Lo Coco, 2018). Consequently, for adolescents who are dealing with a “new” body mentalization and identity construction processes, the body images on SNSs assume great relevance (Pelosi et al., 2014; Franchina and Lo Coco, 2018; Boursier and Manna, 2019). In this regard, social networking activities focused on pictures and visual self-presentation might offer higher perceived control over an individual’s own body image, improving social confidence (Rodgers et al., 2013; Pelosi et al., 2014). On the other hand, such activities might promote appearance-related concerns and potentially problematic monitoring of an individual’s body image and online visual content (Perloff, 2014; Fox and Vendemia, 2016). As previous studies have highlighted, the investment and control over individuals’ own body image in photos pay great attention to picture quality, concerns about self-image shared online (McLean et al., 2015), and strategies in taking and choosing self-pictures before sharing on SNSs (Boursier and Manna, 2019). The asynchronous nature of SNS use might promote the editing utility and an overinvestment of individuals’ body image (Fox and Vendemia, 2016), allowing them to construct and share online the best version of themselves (Fox and Rooney, 2015; Manago et al., 2015; McLean et al., 2016; Casale and Fioravanti, 2017; Boursier and Manna, 2018b; Cohen et al., 2018; Lonergan et al., 2019). This great visual attention directed toward body appearance might trigger behaviors such as body image control and monitoring, potentially related to self-objectification (Vandenbosch and Eggermont, 2012; de Vries and Peter, 2013; Fox and Vendemia, 2016; Butkowski et al., 2019).

In summary, empirical research has confirmed that body image and social networking research fields are strongly connected and rapidly evolving together, highlighting close relationships among appearance-related issues, SNS use, and self-objectification. Within the OBC framework, researchers have mainly focused on the close relationship between body surveillance and SNS use, and only a few studies have examined body shame. No studies have examined appearance control beliefs, which are therefore an understudied aspect of the self-objectification field. Thus, research on the relationship between appearance control beliefs and problematic social networking is still lacking, despite scholarly findings showing that preexisting psychosocial problems, in association with maladaptive cognitions about self, might lead to problematic cognitions, behaviors, and negative outcomes linked to Internet-related activities (Caplan, 2002). Consequently, the present study evaluated the direct and indirect effects of appearance control beliefs and body image control in photos (BICP) upon adolescents’ problematic SNS use, testing the validity of this mediation model across male and female groups. It was expected that appearance control beliefs would influence problematic SNS use and that BICP would mediate the relationship between these variables. Nevertheless, due to the poor and controversial findings concerning appearance control beliefs and the unexplored gender-related differences, a direction for these effects was not specified.

## Materials and Methods

### Participants and Procedure

A total of 693 participants were asked to participate in a survey study. The sample comprised 310 males (45%) and 383 females (55%), aged between 13 and 19 years, with a mean age of 16 years (*SD* = 1.58). Data collection occurred in five different Italian high schools. The parents and school principal of each school were informed of the nature of the research and the measures to be used in the survey, assuring full confidentiality to all participants. Their written consent was provided. General information about the aim of the study was also announced in class. Participation was voluntary, and all participants were informed that they could omit any information they did not wish to give and could withdraw from the study at any time. All students agreed to participate and completed the survey in a classroom setting using their smartphones. The study was approved by the research team’s university research ethics committee and was conducted in accordance with the ethical guidelines for psychological research laid down by the Italian Psychological Association (AIP). No course credits or remunerative rewards were given.

### Measures

#### Socio-Demographic Information and the Amount of Time Spent on SNSs

In this section, information about gender, age, the most used SNSs, and hours per day spent on SNSs was collected. Specifically, participants were asked to answer two items: (i) “Which of these SNSs and Apps do you use mostly,” choosing among WhatsApp, Facebook, Facebook Messenger, Instagram, Snapchat, YouTube, Telegram, Tinder, Tumbler, and Skype and (ii) “How many hours do you spend on SNSs every day,” from 1 (*less than 1 h*) to 8 (*more than 6 h*).

#### Appearance Control Beliefs

The eight-item appearance control beliefs (ACB) subscale of the Italian version of the Objectified Body Consciousness Scale (OBCS; Dakanalis et al., 2017; original English version by McKinley and Hyde, 1996) was used. The ACB subscale evaluates the beliefs by which, given enough effort, physical appearance, body shape, and size can be controlled (e.g. “I think a person can look pretty much how they want to if they are willing to work at it” and “I can weigh what I’m supposed to when I try hard enough”). The items were rated on a seven-point Likert scale ranging from 1 (*strongly disagree*) to 7 (*strongly agree*). Appropriate items are reverse-scored, and an average subscale score was created. In the present study, Cronbach’s α coefficient was good (0.77), lower than values reported by Dakanalis et al. (2017) but comparable with the value reported by McKinley and Hyde (1996).

#### Body Image Control in Photos-Revised

In the present study, the revised (short) version of the BICP-R questionnaire (Boursier and Manna, 2019; for the original version, Pelosi et al., 2014) was used. The BICP-R comprises 16 items rated on a five-point Likert scale, from 1 (*never*) to 5 (*always*), and evaluates adolescents’ photo management and control online and offline, corresponding to five different factors: selfie-related factors (e.g. “I prefer my image as it appears in self-portraits, because I know how to make it look better”), privacy filter behaviors (e.g. “I use privacy filters in order to show photos in which I appear more attractive only to certain people”), positive body image factors (e.g. “I post those photos which I hope will receive praise for my appearance”), sexual attraction factors (e.g. “I have posted provocative photos on Facebook, in order to attract attention to myself”), and negative body image factors (e.g. “I feel awkward if I notice that someone has posted photos that show my body’s defects”). The denomination of BICP-R factors has been modified compared to the previous version of the questionnaire (Boursier and Manna, 2019), to improve their intelligibility. Similar to a previous study that used the BICP-R (Boursier and Manna, 2019), in the present study, Cronbach’s α value for the scale was very good (0.82).

#### Generalized Problematic Internet Use Scale 2

In the present study, the Italian version of Generalized Problematic Internet Use Scale 2 (GPIUS2) (Fioravanti et al., 2013) was used. The GPIUS2 (Caplan, 2010) is a 15-item scale rated on a seven-point Likert scale, from 1 (*strongly disagree*) to 7 (*strongly agree*), and assesses the degree of generalized problematic Internet use, examining five factors: preference for online social interactions (e.g. “I prefer communicating with people online rather than face-to-face”), mood regulation (e.g. “I have used the Internet to make myself feel better when I was down”), cognitive preoccupation (e.g. “I think obsessively about going online when I am offline”), compulsive Internet use (e.g. “I have difficulty controlling the amount of time I spend online”), and negative outcomes (e.g. “My Internet use has created problems for me in my life”). As in a previous study by Casale and Fioravanti (2017), for the purposes of this study, the word “Internet” was replaced by “social network sites” to explore potential problematic social networking among adolescents (e.g. “I have used SNSs to make myself feel better when I was down”). In the present study, Cronbach’s α was very good (0.88) and Cronbach’s α values for each subscale were 0.69 (preference for online social interactions), 0.73 (mood regulation), 0.81 (cognitive preoccupation), 0.82 (compulsive SNS use), and 0.75 (negative outcomes).

### Statistical Analysis

Descriptive statistics were performed using the Statistical Package for Social Sciences (SPSS Version 23 for Windows) and it was used to assess the means, standard deviation of the variables, and confidence interval of means (CI: 95%). Independent *t*-tests were used to assess gender differences, and the magnitude of the differences were evaluated with effect sizes (Cohen’s *d*). Path analyses within structural equation modeling (SEM) were used to test the proposed mediation model. To evaluate the overall model goodness of fit, several indexes were used: the comparative fit index (CFI) and the Tucker–Lewis fit index (TLI), which are indices related to the total variance accounted for by the model and where values higher than 0.90 are desired (Bentler, 1990) root mean square error approximation (RMSEA), which is related to the variance of residuals and for which values below 0.08 are recommended (Browne and Cudeck, 1993); and the standardized root mean square residuals (SRMR) for which values below 0.08 are considered a good fit (Kline, 2015). The Satorra–Bentler χ^2^ difference test (ΔSB χ^2^) was used to test the relative fit of nested models (Satorra, 2000). When the more constrained model was rejected, a gradually less restrictive model of partial invariance was tested. All SEM analyses were performed utilizing MPlus 8 (Muthén & Muthén, Los Angeles, CA, United States).

## Results

### Descriptive Statistics

Among the participants, the most popular and used SNSs were WhatsApp (99%), Instagram (92%), YouTube (80%), and Facebook (70%). Descriptive analyses and gender differences are reported in Table 1. Statistically significant differences between males’ and females’ scores were found. Girls reported higher mean scores in hours per day spent on SNSs, appearance control beliefs, selfie-related factors, privacy filter behaviors, positive body image factors, negative body image factors, mood, cognitive preoccupation, and compulsive SNS use. On the contrary, boys showed higher mean scores in sexual attraction factors and negative outcomes. The effect sizes (Cohen’s *d*) were small for privacy filter behaviors, positive body image factors, negative body image factors, mood regulation, and cognitive preoccupation. Medium effect sizes were found for compulsive SNS use and negative outcomes. Finally, relevant effect sizes were found for body control beliefs, selfie-related factors, and sexual attraction factors. Bivariate correlations between all variables are shown in Table 2. Negative correlations were generally found between appearance control beliefs and BICP and problematic SNS use in both male and female samples.

**TABLE 1:** Means, standard deviations (*SD*), confidence intervals (CI), *t*-test, and effect sizes (Cohen’s *d*) for both genders.

	Total sample	Males	Females		
	Mean (*SD*) [95% CI]	Mean (*SD*) [95% CI]	Mean (*SD*) [95% CI]	*t*	*d*
Hours per day spent on SNSs	3.40 (1.209) [3.32–3.49]	3.08 (1.230) [2.95–3.22]	3.66 (1.127) [3.55–3.78]	6.456***	*0.49*
OBCS appearance control beliefs	4.91 (0.785) [4.852–4.968]	4.676 (0.869) [4.575–4.769]	5.099 (0.651) [5.031–5.164]	7.313***	0.56
BICP selfie-related factors	2.712 (1.093) [2.630–2.793]	2.329 (1.043) [2.215–2.450]	3.021 (1.035) [2.919–3.122]	8.729***	0.66
BICP privacy filter behaviors	1.70 (1.078) [1.615–1.777]	1.597 (1.062) [1.482–1.71]	1.779 (1.085) [1.658–1.886]	2.224*	0.17
BICP positive body image factors	2.775 (0.845) [2.713–2.836]	2.676 (0.883) [2.582–2.773]	2.854 (0.806) [2.774–2.930]	2.780**	0.21
BICP sexual attraction factors	1.68 (1.143) [1.604–1.761]	2.027 (1.380) [1.871–2.184]	1.398 (0.805) [1.316–1.482]	7.488***	0.57
BICP negative body image factors	3.175 (1.191) [3.075–3.263]	3.011 (1.272) [2.868–3.155]	3.308 (1.105) [3.200–3.427]	3.291**	0.25
PSNSU preference for online social interactions	2.467 (1.504) [2.343–2.583]	2.460 (1.498) [2.290–2.632]	2.472 (1.510) [2.32–2.621]	0.100^n.s.^	0.01
PSNSU mood regulation	3.299 (1.767) [3.176–3.425]	3.04 (1.775) [2.844–3.256]	3.509 (1.734) [3.33–3.686]	3.506***	0.27
PSNSU cognitive preoccupation	3.242 (1.849) [3.097–3.387]	2.967 (1.766) [2.785–3.179]	3.465 (1.887) [3.286–3.655]	3.555***	0.27
PSNSU compulsive social network site use	3.306 (1.943) [3.162–3.453]	2.869 (1.785) [2.672–3.069]	3.661 (1.996) [3.470–3.86]	5.443***	0.41
PSNSU negative outcomes	1.965 (1.312) [1.877–2.061]	2.224 (1.315) [2.078–2.37]	1.756 (1.273) [1.630–1.889]	4.735***	0.36

OBCS, Objectified Body Consciousness Scale; BICP, body image control in photos; PSNSU, problematic social network site use. * *p* < 0.05; ** *p* < 0.01; *** *p* < 0.001; n.s., non-significant.

**TABLE 2:** Bivariate correlations between all variables.

	1	2	3	4	5	6	7	8	9	10
1. OBCS appearance control beliefs	–	−0.113*	−0.165**	−0.137**	−0.053	−0.144**	−0.213**	−0.173**	−0.225**	−0.227**
2. BICP selfie-related factors	−0.148**	–	0.259**	0.461**	0.292**	0.565**	0.123*	0.310**	0.456**	0.394**
3. BICP privacy filter behaviors	−0.077	0.355**	–	0.277**	0.274**	0.249**	0.158**	0.248**	0.134**	0.170**
4. BICP positive body image factors	−0.420**	0.350**	0.282**	–	0.241**	0.554**	0.174**	0.343**	0.319**	0.301**
5. BICP sexual attraction factors	−0.407**	0.301**	0.228**	0.426**	–	0.261**	0.176**	0.189**	0.252**	0.231**
6. BICP negative body image factors	−0.344**	0.527**	0.235**	0.463**	0.417**	–	0.206**	0.312**	0.413**	0.342**
7. PSNSU preference for online social interactions	−0.150**	0.238**	0.311**	0.146*	0.234**	0.223**	–	0.414**	0.344**	0.337**
8. PSNSU mood regulation	0.116*	0.297**	0.315**	0.080	0.031	0.221**	0.515**	–	0.401**	0.455**
9. PSNSU cognitive preoccupation	−0.021	0.343**	0.346**	0.188**	0.055	0.224**	0.425**	0.591**	–	0.784**
10. PSNSU compulsive social network site use	0.038	0.289**	0.360**	0.095	0.024	0.206**	0.488**	0.640**	0.710**	–

Males’ data below the diagonal, females’ data above the diagonal.OBCS, Objectified Body Consciousness Scale; BICP, body image control in photos; PSNSU, problematic social network site use. * *p* = 0.05; ** *p* = 0.01.

### Mediation Analysis

The mediated effect of appearance control beliefs on problematic SNS use via BICP was tested. An unconstrained model in which all paths were allowed to freely vary was tested across male and female groups. The model produced an inadequate fit to the data, MLR χ^2^(48) = 264.139, *p* < 0.001; CFI = 0.90; TLI = 0.76; RMSEA = 0.114, 90% CI [0.101–0.128]; SRMR = 0.087. The subsequent fully constrained model showed a little improvement of the model fit, MLR χ^2^(82) = 324.349, *p* < 0.001; CFI = 0.88; TLI = 0.84; RMSEA = 0.092, 90% CI [0.082–0.103]; SRMR = 0.091. Nevertheless, comparing the fit of the unconstrained model to the fit of a fully constrained model, the ΔSB χ^2^ indicated that groups were already different: ΔSB χ^2^(34) = 60.21, *p* = 0.004. Thus, invariance has not been established.

**FIGURE 1:**
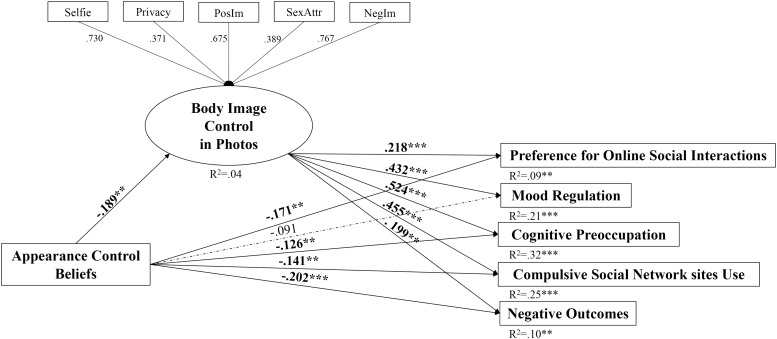
The overall mediation model with standardized path coefficients and the explained variance of the endogen variables (*R*^2^). The mediator variable is a latent variable. Simple arrows: significant path coefficients, dotted arrows: non-significant path coefficients. Selfie, selfie-related factors subscale; Privacy, privacy filter behaviors subscale; PosIm, positive body image factors subscale; SexAttr, sexual attraction factors subscale; NegIm, negative body image factors subscale. ^∗∗^*p* < 0.01; ^∗∗∗^*p* < 0.001.

Consequently, the mediation model was tested on both male and female independent samples. The mediation model on the male group showed a quite inadequate fit to the data, MLR χ^2^(29) = 107.742, *p* < 0.001; CFI = 0.92; TLI = 0.85; RMSEA = 0.094, 90% CI [0.075–0.113]; SRMR = 0.072. Differently, the mediation model on the female sample showed an optimal fit to the data: MLR χ^2^ = 66.144, *p* < 0.001; CFI = 0.97; TLI = 0.94; RMSEA = 0.058, 90% CI [0.039–0.076]; SRMR = 0.040. According to the results (Figure 1), appearance control beliefs have a significant direct negative effect on BICP, preference for online social interactions, cognitive preoccupation, compulsive SNS use, and negative outcomes. BICP was significantly and strongly associated with problematic SNS use, with direct effects on preference for online social interactions, mood regulation, cognitive preoccupation, compulsive SNS use, and negative outcomes.

In relation to the indirect effects, all paths were statistically significant: (i) appearance control beliefs → BICP → preference for online social interactions (β = −0.041; *p* < 0.05), (ii) appearance control beliefs → BICP → mood regulation (β = −0.081; *p* < 0.01), (iii) appearance control beliefs → BICP → cognitive preoccupation (β = −0.099; *p* < 0.01), (iv) appearance control beliefs → BICP → compulsive SNS use (β = −0.086; *p* < 0.01), and (v) appearance control beliefs → BICP → negative outcomes (β = −0.037; *p* < 0.05). The full model explained 9% of the total variance of preference for online social interactions, 21% for mood regulation, 32% for cognitive preoccupation, 25% for compulsive SNS use, and 10% for negative outcomes.

## Discussion

The present study primarily focused on the understudied construct of appearance control beliefs, contributing to the OBC research field and the ongoing debate concerning predictive factors in problematic SNS use. More specifically, a mediation model was tested to explore the predictive role of appearance control beliefs on problematic SNS use via BICP.

Differently from previous findings (e.g. McKinley, 1998; John and Ebbeck, 2008; Dakanalis et al., 2017), in which no statistically significant gender differences concerning appearance control beliefs have been reported, girls in the present study showed higher rates of appearance control beliefs than boys with a relevant effect size. This result appears to fit OBC theory’s underlying assumption that females, more than males, internalize the belief that they are responsible for their physical appearance and that, given enough effort, they can control it complying with cultural standards.

Overall, in the present study, adolescents showed a preference for WhatsApp (an app that promotes the exchange of messages, pictures, and videos) as well as for body image-focused SNSs (Instagram, YouTube, and Facebook). However, in accordance with previous findings (Griffiths et al., 2014; Andreassen et al., 2017; Boursier and Manna, 2019), the present study also showed higher engagement by girls relating to time spent on SNSs and BICP, investing more time than boys in creating self-portraits as a way to express their identity and to manage own positive and negative images, promoting their best self-presentation and applying privacy restrictions to moderate relational exchanges via photos. On the contrary, boys used greater body image control to improve their sexual attractiveness, confirming the males’ great attention for sexual aspects of online body images that might promote sexual exploration experiences (Boursier and Manna, 2018b). Moreover, in terms of problematic social networking, female adolescents were significantly more likely than males to use SNSs to regulate their mood states, with higher cognitive preoccupation and poorly self-regulated SNS use. These findings appeared in line with several previous studies that found a strong association between females’ engagement in social media use and depressive mood, low self-esteem, and other psychological distress, leading to their greater problematic social networking (McCrae et al., 2017; Nowland et al., 2018; Raudsepp and Kais, 2019). However, the negative outcomes due to problematic social networking appeared to affect more boys than girls, likely due to males’ higher attention for sexual aspects of online body image-related activities and engagement in online sexual behaviors (Jonsson et al., 2014; Bianchi et al., 2018; Boursier and Manna, 2018b) and/or due to a higher online disinhibition (Casale et al., 2015) despite other studies finding that females were more engaged in online self-disclosure (Schouten et al., 2007). No statistically significant difference between girls’ and boys’ preferences for online social interactions was found. In line with Boursier and Manna (2019), the present study found a positive correlation between males’ and females’ BICP and adolescents’ problematic SNS use. Interestingly, appearance control beliefs negatively co-occurred with BICP and problematic social networking, especially among females.

Concerning the mediation model, invariance was not established. Subsequently, the mediation model was tested on independent male and female samples, being significant only among female adolescents. Different interpretations of the current findings are possible. Firstly, they might confirm the OBC’s assumption that females, more than males, consider themselves responsible for how they look and that, given enough effort, they can control their appearance to satisfy cultural standards (McKinley and Hyde, 1996). Internalizing the outside observer’s perspective on body and cultural appearance standards, females might perceive them as a personal choice that in turn promotes beliefs in appearance controllability (McKinley and Hyde, 1996). Moreover, the large effect size of gender-related difference in appearance control beliefs might confirm this interpretation. Differently, the lack of invariance might suggest that male and female adolescents involved in the present study differently perceived the content of the appearance control beliefs items. Finally, these controversial results might confirm Moradi and Varnes’ (2017) findings about the uncertain belonging of appearance control beliefs to the OBC framework, suggesting further investigation, refinement, and conceptualization.

Nevertheless, the mediation model confirmed the expected effect of appearance control beliefs on problematic social networking, with the mediating effect of BICP. More specifically, interestingly, appearance control beliefs showed a direct and negative effect on BICP, confirming previous empirical findings in which believing in control over one’s own physical appearance leads to a decrease of body monitoring and feelings of shame toward one’s own body (Taylor, 1989; Noser and Zeigler-Hill, 2014) and an increase of healthy behaviors (Sinclair, 2010). Similarly, in the present study, female adolescents who believed they could control their own body appearance might become less vigilant about their body image in photos, picture quality, their self-image promoted online, and strategies for taking, choosing, and editing their shared photos online (Manago et al., 2015; McLean et al., 2015; Boursier and Manna, 2019). It is likely, adolescents who believe they can control their own appearance might feel more positive regarding their bodies (McKinley and Hyde, 1996; John and Ebbeck, 2008) show a greater sense of competence (Sinclair and Myers, 2004). Moreover, beliefs that body appearance can be controlled, as expected, directly predicted problematic social networking. Specifically, appearance control beliefs negatively predicted adolescents’ preference for online social interactions, cognitive preoccupation, compulsive SNS use, and negative outcomes (likely assuming a protective function). It is likely, girls who believe they can control their own appearance and thus feel more positive regarding their bodies (McKinley and Hyde, 1996; John and Ebbeck, 2008) do not prefer online contexts for relational exchanges, with consequent less SNS use-related cognitive preoccupation, compulsive use, and negative outcomes. Furthermore, according to previous studies in which beliefs of control over own life and appearance have been found as a means of relieving stress and anxiety situations (McKinley and Hyde, 1996; McKinley, 1999; Sinclair and Myers, 2004), these findings might explain why females in the present study did not seem to use SNSs to regulate their mood. Additionally, appearance control beliefs confirmed their negative (and likely protective) effect on adolescents’ problematic social networking also through the reduced engagement in BICP. In this regard, previous studies have highlighted that problematic Internet-related activities and consequent negative outcomes were related to the perceived utility of online contexts for providing greater control compared to face-to-face environments (Fioravanti et al., 2012; Casale et al., 2015). It is likely, the present study suggests that girls who believe they can control their own appearance do not perceive or do not need this SNS benefit. In this regard, the present study seems to disagree with the chain relationships in which the perceived control over personal information might enhance individuals’ confidence in managing it in online contexts, reducing the perception of SNS-related risks (Niemz et al., 2005; Joinson et al., 2010; Krasnova et al., 2010; Taddei and Contena, 2013). Moreover, appearance control beliefs appear different from positive metacognitions that promote the engagement in problematic behaviors (Spada et al., 2007, 2015; Casale et al., 2016). On the contrary, according to previous studies within the OBC framework (McKinley and Hyde, 1996; McKinley, 1998, 1999; Sinclair and Myers, 2004; Laliberte et al., 2007; Moradi, 2010; Sinclair, 2010), the present findings seem to confirm the involvement, into appearance control beliefs, of a sense of agency, sense of competence, perceived generalized controllability over life events, and locus of control, which in turn might promote healthy behaviors, body satisfaction, and psychological well-being (McKinley and Hyde, 1996; McKinley, 1999; Sinclair and Myers, 2004; John and Ebbeck, 2008; Crawford et al., 2009; Sinclair, 2010; Noser and Zeigler-Hill, 2014).

Therefore, it appears that perceived control affects behaviors and emotions (Schall et al., 2016) and, likely, believing in the ability to control one’s own body appearance may be seen as a skill, improve perceived self-efficacy, and contribute to physical self-worth (John and Ebbeck, 2008). Nevertheless, as Crawford et al. (2009) stated, females’ beliefs that appearance control is in their own hands, which might lead to accepting both negative and positive judgments over their body images, are warranted, often fueling self-blame for perceived failure of control and leading to other negative outcomes (i.e. excessive exercise, dietary restrictions, and marginalization) (Schall et al., 2016). Therefore, how girls interact with their own body and photos prior to posting on SNSs appears to be strongly associated with problematic social networking (Cohen et al., 2018), especially during adolescence, when social reward and peer approval are pivotal motivators of adolescents’ behavior (Foulkes and Blakemore, 2016; Bell et al., 2018).

The present study’s findings provided some novel and previously unreported issues. The findings demonstrated the understudied association between appearance control beliefs and monitoring of body image in the online environment. More specifically, females who feel they can control their own body image appear to decrease strategies to control their body image in photos. Furthermore, the present study showed the unexplored effect of appearance control beliefs and BICP upon problematic SNS use. Therefore, firstly, these findings contribute to the ongoing debate concerning predictive and protective factors related to problematic SNS use, confirming the pivotal role of body image-related issues in relation to social networking and its misuse. Secondly, the present study contributes to the OBC research field and the debated (and controversial) role of appearance control beliefs within this framework. The inconsistent mix of positive, negative, or non-significant relationships between appearance control beliefs and other indicators of OBC (McKinley and Hyde, 1996; McKinley, 1998, 1999; Parsons and Betz, 2001; Moradi and Varnes’, 2017) has led to a gradual disregard of appearance control belief implications with regard to body image and social media use issues. Nevertheless, as Moradi and Varnes’ (2017) stated, rather than abandoning appearance control beliefs, further research is needed to refine this multidimensional construct and operationalize it by investigating understudied dimensions such as sense of agency, locus of control, and personal competence (McKinley and Hyde, 1996; McKinley, 1998, 1999; Parsons and Betz, 2001; Sinclair and Myers, 2004; John and Ebbeck, 2008; Crawford et al., 2009; Noser and Zeigler-Hill, 2014; Moradi and Varnes’, 2017).

Some limitations of the present study also need to be addressed. Firstly, the participants involved in the study came from a specific (Italian) cultural context, and these cross-sectional data limited the ability to formally test causality. Indeed, it is plausible to suppose that problematic SNS use and appearance control beliefs might mutually affect and reinforce each other, according to Strelan and Hargreaves’ (2005) circle of self-objectification concerning the bidirectional nature of the SNS use–self-objectification relationship. Secondly, the study used a self-report survey, and its potential method biases are well known. Moreover, despite the participants reporting a great preference for images-based SNSs, the present study did not focus on specific photographic SNSs, such as Instagram or Snapchat. Future research should explore the relationships between appearance control beliefs and specific body image-based SNSs, likely in association with other appearance-related issues (for example, body dissatisfaction and photo-editing). Finally, the present study explored only a small number of variables in relation to the complex constructs of OBC and problematic SNS use. Thus, future research could consider other variables such as body image-related issues, personality traits, and peer-to-peer friendships. However, the findings presented here might contribute to future research and intervention programs. Based on the findings of previous studies (i.e. Sinclair, 2010; McLean et al., 2016; Fardouly et al., 2018), media literacy interventions are needed to educate adolescents about their real body image, their feelings and self-efficacy about physical appearance, culturally and peer-to-peer promoted body standards, and their sharing of photos on SNSs.

## Data Availability Statement

The datasets generated for this study are available on request to the corresponding author.

## Ethics Statement

The studies involving human participants were reviewed and approved by the University of Naples Federico II Research Ethics Committee. Written informed consent to participate in this study was provided by the participants’ legal guardian/next of kin.

## Author Contributions

VB designed the study and revised it critically, and contributed to writing the final version of manuscript. VB and FG led the literature search and interpretation of data. FG contributed to the data collection and statistical analysis. FG wrote a first draft of manuscript. MG revised the whole work critically for important intellectual content. All authors read and approved the final version of work to be published and agreed to be accountable for all aspects of the work, ensuring that questions as to the accuracy of any part of the work are appropriately investigated and resolved.

## Conflict of Interest

The authors declare that the research was conducted in the absence of any commercial or financial relationships that could be construed as a potential conflict of interest.
